# Highlight: Unpicking the Secrets of the “Great Speciator”

**DOI:** 10.1093/gbe/evv181

**Published:** 2015-09-29

**Authors:** Danielle Venton

Nestled in the South Pacific archipelago of New Caledonia, the island of Lifou is home to a famous bird. To biologists interested in biodiversity, white-eyes (genus *Zosterops*) are the masters of spawning diversity. Evolutionary biologists Ernst Mayr and Jared Diamond dubbed the birds the “great speciator.”

White-eyes are remarkable for appearing in so many varieties. Their order, the passerines, accounts for half of all the species richness found within birds. Among passerines *Zosterops *is the most diverse of genera, with about 80 described species found in Africa, Australasia, and the South Pacific islands.

The birds excel at colonizing islands. Three sympatric species exist on Lifou, which spans just about 1,200 km^2^, providing scientists with an ideal study model.

“The island is incredible. You arrive and find three endemic species of *Zosterops *living in the same habitat, which is very unusual,” says Luis Valente, coauthor of a recent study analyzing why white-eyes do so well at speciation. “From an evolutionary biologist’s perspective, it’s a really exciting environment.”

Previous work has established that these are rapidly evolving birds, but little has been done to peek at the underlying mechanisms. Valente and colleagues were recently the first to sequence and publish a reference genome of *Zosterops*. They use it as a model against which they can test hypothesis regarding why white-eyes diversified so abundantly compared with other birds. Their work can be found in the XXXXX issue of *Genome Biology Evolution *(Cornetti et al. 2015).

“The idea is that if we look at these species that have been evolving quite rapidly, we can get perhaps more information about the processes that create biodiversity,” says Valente.

Valente and coauthors compared the genomes of the three species endemic to Lifu with each other and then expanded their view to compare the genome of *Zoetropes *as a whole with the genomes of other bird groups (ones which do not diversify as fast and whose genomes have already been published).

“The most interesting thing,” Valente says, “is that all of the results went in the direction that we’d expect.” (Such is not always the case in similar investigations, Valente explains.) The team hypothesized that *Zoetropes* would have a relatively higher number of substitutions, because the changes in their DNA sequences are very rapid. And so it was. They also found a very high number of gene duplications, implying that there are more new genes being formed in the *Zoetropes* lineage compared with other birds. They also find many genes under positive selection, indicating that the genome of *Zoetropes *is quite evolutionarily flexible.

“When you think of the theory of how things evolve very rapidly, this is exactly what we find in the genomes of *Zoetropes*,” says Valente.

Valiant cautions the work is preliminary and theoretical, but the idea is, he says, if a species has a genome that is very flexible and changes so that new genes are easily formed or different features of the phenotypes appear (rapidly changing wings, for instance) then this can potentially lead to the formation of new species faster than normal.

The work might be preliminary, but it is interesting, says Walter Jetz, an evolutionary biologist who was not involved in the study. He would like to see genomes from other species given the same analytical treatment, but says “the work provides a nice demonstration of the sorts of new insights gained from combining genomic among and within species in an explicit spatial and biogeographic context.”

Prior to publishing the results of their analysis, Valente says that they were eager to release the reference genome of silvereyes for others to use.

“It’s quite exciting to be able to produce the first genome for Zoestrops,” he says, “because it has been a model system for quite a few years and the genome has been in quite some demand.”

Asking questions about how genomic characteristics underpin diversification is primarily a theoretical exercise—one that works at solving a fundamental question in biology. Yet Valente thinks this could also be a useful tool for conservationists intent on preserving biodiversity. Though white-eyes are not endangered the method “could show where the genetic diversity is and which populations are priorities for conservation,” he says. “It’s important information to have, but conservationists generally don’t do it because it is very expensive.”

In all likeliness, genomic sequencing will continue to become more accessible in the future, and will help cast light on how biodiversity is generated and maintained. If we understand that, Valente says, “perhaps we can predict what might happen to the equilibrium of species if we disturb habitats. It’s quite a wide fundamental question really.” 
